# In Vivo Validation of Elekta's Clarity Autoscan for Ultrasound-based Intrafraction Motion Estimation of the Prostate During Radiation Therapy

**DOI:** 10.1016/j.ijrobp.2018.04.008

**Published:** 2018-11-15

**Authors:** Alexander Grimwood, Helen A. McNair, Tuathan P. O'Shea, Stephen Gilroy, Karen Thomas, Jeffrey C. Bamber, Alison C. Tree, Emma J. Harris

**Affiliations:** ∗ Division of Radiotherapy and Imaging, The Institute of Cancer Research and Royal Marsden Hospital Trust, Sutton, UK; † North West Cancer Centre, Altnagelvin Area Hospital, Londonderry, Ireland

## Abstract

**Purpose:**

Our purpose was to perform an in vivo validation of ultrasound imaging for intrafraction motion estimation using the Elekta Clarity Autoscan system during prostate radiation therapy. The study was conducted as part of the Clarity-Pro trial (NCT02388308).

**Methods and Materials:**

Initial locations of intraprostatic fiducial markers were identified from cone beam computed tomography scans. Marker positions were translated according to Clarity intrafraction 3-dimensional prostate motion estimates. The updated locations were projected onto the 2-dimensional electronic portal imager plane. These Clarity-based estimates were compared with the actual portal-imaged 2-dimensional marker positions. Images from 16 patients encompassing 80 fractions were analyzed. To investigate the influence of intraprostatic markers and image quality on ultrasound motion estimation, 3 observers rated image quality, and the marker visibility on ultrasound images was assessed.

**Results:**

The median difference between Clarity-defined intrafraction marker locations and portal-imaged marker locations was 0.6 mm (with 95% limit of agreement at 2.5 mm). Markers were identified on ultrasound in only 3 of a possible 240 instances. No linear relationship between image quality and Clarity motion estimation confidence was identified. The difference between Clarity-based motion estimates and electronic portal–imaged marker location was also independent of image quality. Clarity estimation confidence was degraded in a single fraction owing to poor probe placement.

**Conclusions:**

The accuracy of Clarity intrafraction prostate motion estimation is comparable with that of other motion-monitoring systems in radiation therapy. The effect of fiducial markers in the study was deemed negligible as they were rarely visible on ultrasound images compared with intrinsic anatomic features. Clarity motion estimation confidence was robust to variations in image quality and the number of ultrasound-imaged anatomic features; however, it was degraded as a result of poor probe placement.

**Summary**

This article describes an in vivo validation of the Elekta Clarity Autoscan system for estimating intrafraction prostate motion during radiation therapy. Intrafraction motion was estimated using Autoscan. These 3-dimensional motion estimates were used to estimate intraprostatic fiducial marker positions on 2-dimensional portal images. Estimated positions were compared with the actual portal-imaged marker positions. The Clarity system monitored intrafraction motion with an accuracy of 0.6 mm, which is comparable with other motion-monitoring systems used in radiation therapy.

## Introduction

Prostate cancer radiation therapy (RT) is effective at achieving long-term control of the tumor but at the expense of potential side effects in surrounding organs at risk, such as the rectum (1). Moderate and extreme hypofractionation regimens have been studied to optimize therapeutic response while minimizing toxicity in normal tissue 2, 3. The efficacy of moderate hypofractionated prostate RT has been demonstrated in recent trials, with growing evidence of the effectiveness of more extreme hypofractionation delivered using stereotactic body radiation therapy (SBRT), leading to rapid adoption of prostate SBRT in a number of countries 2, 4, 5. The implications of intrafraction motion increase with hypofractionation, and hence there is growing interest in intrafraction motion monitoring.

SBRT typically uses 5 fractions, with small planning target volume (PTV) margins between 2 and 5 mm. The magnitude of intrafraction motion as a percentage of treatment time was shown to be up to 14% for displacements > 3 mm and up to 3% for displacements > 5 mm, with displacements > 10 mm observed in patients undergoing intensity modulated radiation therapy (IMRT) 6, 7, 8, 9. Prostate motion has been characterized previously as a stochastic process, such as a random walk, where positional variance increases over time (8). The motion is largely attributed to transitory gas and filling of the rectum, but bladder filling and femoral head rotation can also contribute. Regular enemas and bladder-filling protocols are routinely used during RT to assist consistent patient setup and reduce motion, but these alone cannot achieve the accuracy necessary for SBRT (10).

Potentially suitable systems have been developed to monitor prostate position throughout treatment. CyberKnife (Accuray, Sunnyvale, CA) tracks radiopaque intraprostatic fiducial markers 3-dimensionally using stereoscopic kilovoltage x-ray imaging 11, 12. The kilovoltage intrafraction monitoring (KIM) system estimates 3-dimensional (3D) prostate motion by imaging implanted fiducial markers using a standard C-arm linear accelerator (linac) kilovoltage imager 13, 14. Calypso (Varian Medical Systems, Palo Alto, CA) and RayPilot (Micropos, Gothenburg, Sweden) use electromagnetic transponders independent of treatment platform. Calypso transponders are implanted instead of fiducial markers, and an external receiver detects their position at a rate of 25 Hz 15, 16. RayPilot incorporates a single catheter-based transponder, deployed via the urethra to the prostate, with a 30-Hz update frequency 17, 18. Other systems, such as the MRIdian (ViewRay, Bedford, OH) and MR-linac (Elekta, Stockholm, Sweden) integrate magnetic resonance imaging with RT systems to facilitate adaptive treatment and motion management 19, 20.

The Elekta Clarity Autoscan system uses transperineal ultrasound (TPUS) without relying on fiducial markers or extensive hardware installation. Ultrasound (US) imaging has shown promise for patient setup verification and interfraction motion management (9). The modality is particularly suited to prostate RT in which the clinical treatment volume (CTV) is unobstructed by bone or air and moves relative to bony anatomy (21). The Clarity system could improve care for patients in 2 key ways: First, avoiding the risks, discomfort, and inconvenience of fiducial insertion would be beneficial to patients and health care economies. Second, the Clarity system is one of the few systems that can monitor intrafraction motion of the prostate on a conventional C-arm linac. Prostate motion during the course of 1 fraction can be enough to result in geographic miss, even if patients are set up correctly immediately prior to “beam on” 6, 22. The clinical implications of any motion become increasingly apparent as we move toward extreme hypofractionation. A noninvasive, accurate method of intrafraction motion monitoring would improve the accuracy of delivery, reducing the chance of treatment failure owing to geographic miss.

The high accuracy of Autoscan has been demonstrated in vitro 23, 24. Lachaine and Falco (23) reported a mean error ≤ 0.2 mm and standard deviation ≤ 0.4 mm for an anthropomorphic pelvic phantom moving with an amplitude ≤ 20 mm. Fast et al (24) implemented dynamic multileaf collimator (dMLC) tracking guided by the Clarity system. A root-mean-square error of 0.7 mm was reported for 3D Autoscan monitoring of a moving quality control phantom using prostate motion traces.

The purpose of this study was to perform an in vivo validation of Autoscan in patients receiving IMRT for prostate cancer. Performance regarding intrafraction motion estimation was evaluated by comparing Clarity-measured prostate motion with prostate motion determined using implanted fiducial markers and electronic portal imaging (EPI).

## Methods and Materials

### Clarity study

This study was conducted as part of the Clarity-Pro clinical trial (NCT02388308). Prostatectomy patients were ineligible for the trial. Patients consented to undergo TPUS at treatment preparation, at verification, and during radiation delivery. The study was approved by the Surrey and SE Coast Regional Ethics Committee in the United Kingdom.

### Treatment preparation

Three cylindrical intraprostatic gold markers (FlexiMarc; Cortex Manufacturing, Lake Stevens, WA), 1 mm in diameter and 3 mm in length, were implanted under transrectal US guidance in all patients at least 1 week before simulation (SIM) computed tomography (CT). Patients received a CT scan (Brilliance Big Bore; Philips, Amsterdam, The Netherlands) and 3D TPUS SIM scan while positioned using the Clarity Autoscan probe kit incorporating knee rests and a TPUS probe fixed to the couch by a baseplate. Probe positioning and imaging were optimized by manually reviewing live B-mode US images prior to CT. Ideally, the prostate appeared centrally in the US volume, with the bladder, rectum, penile bulb, and pubic symphysis also visible. Probe- and baseplate-indexed positions were recorded to aid reproducible setup on treatment. The CT acquisition parameters were 140 kV, slice thickness of 1.5 mm, and pixel size of 1 mm. Both the CT and treatment rooms incorporated a ceiling-mounted infrared camera to track the probe in room coordinates, enabling US volume registration to the isocenter. SIM was acquired immediately prior to CT.

Adhering to local clinical practice, we followed bladder-filling and rectal-clearing protocols to mitigate anatomic variation during treatment. Patients drank 350 mL of water 1 hour prior to SIM and treatment. Patients underwent enemas 2 consecutive days before SIM, 1 hour prior to CT, 2 consecutive days before treatment, and for the first 10 treatment days. Rectal filling was measured on CT SIM. Two patients with a rectal diameter > 4 cm from anterior to posterior and 3.5 cm from left to right were scheduled for a rescan, including SIM, after 2 days of additional enemas.

Five- or three-field step-and-shoot IMRT plans were devised in ADAC Pinnacle (Philips) for the Elekta Synergy linac with Agility multileaf collimator (MLC), delivering 60 Gy in 20 fractions or 74 Gy in 37 fractions 25, 26. Three shrinking PTV margins from 6 mm to 0 mm, incorporating seminal vesicles as clinically indicated, were used (24). The CT image, treatment contours (CTV, rectum, bladder, and penile bulb), and plan were imported into Clarity Automated Fusion and Contouring software where SIM and CT scans were registered. A reference positioning volume (RPV) was contoured on SIM within the CTV to define a reference template for Autoscan motion estimation during treatment, the template being the RPV grown by 2 mm (23).

### Treatment and imaging

The patient was set up on the treatment couch with tattoo markers aligned to room lasers. Live TPUS images were compared with SIM to assist with probe positioning.

**Fig. 1:**
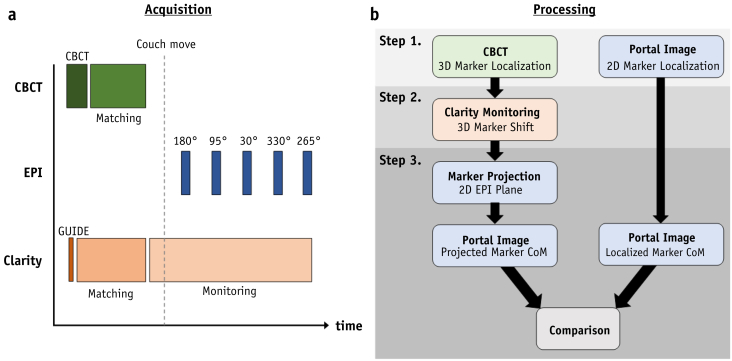
a, Imaging workflow timeline for a typical 5-field intensity modulated radiation therapy fraction. b, Three-step image processing workflow depicting (*1*) manual marker localizations, (*2*) Autoscan-based localization, and (*3*) projection and comparison of marker centers of mass (CoM). CBCT = cone beam computed tomography; EPI = electronic portal imaging; 2D = 2-dimensional; 3D = 3-dimensional.

Prostate position and requisite couch moves were determined on treatment by matching cone beam computed tomography (CBCT) to planning CT using a mask registration incorporating the prostate and markers (XVI; Elekta). Figure 1 a depicts the treatment imaging workflow. A single 3D ultrasound (Guide) scan was acquired in approximately 3 seconds at the start of the 60-second CBCT acquisition. During CBCT acquisition and registration, a copy of the RPV contour, called the guidance positioning volume (GPV), was manually registered on Guide to match the SIM prostate position. Autoscan monitoring was started prior to couch movement, and intrafraction motion estimation data were continuously acquired until treatment ended.

Monitoring has been described previously by Lachaine and Falco (23) and O'Shea et al (27). Autoscan continuously and automatically acquires 3D image data. A GPV template is defined on the first monitoring image and compared with all subsequent frames using intensity-based cross correlation. Motion estimates incorporate translation and rotation and are updated at a rate of approximately 3 Hz, depending on acquisition parameters. An associated estimation confidence factor (*C*) is generated by a proprietary algorithm (28). A number of parameters are used to calculate *C*, including the maximum correlation score, the similarity metric between the GPV template and a region 1 mm from the search location, and the minimum and maximum correlation scores for subregions within the GPV. Cine-mode EPI images (iView; Elekta) were acquired. The EPI panel detection area was 41 × 41 cm, comprising 1024 × 1024 pixels. Image pixel size was 0.25 mm at the isocenter.

### Comparison of Autoscan- versus marker-measured motion

Prostate motion estimates were calculated by identifying the initial 3D marker positions and applying translations recorded by Autoscan. The Autoscan-predicted marker positions were projected along the treatment beam path onto the 2-dimensional (2D) EPI plane and compared with the actual location of portal-imaged markers. Image processing and analysis are illustrated in 3 steps (Fig. 1 b).

#### Step 1: Prostate localization using CBCT and portal images

Three observers manually identified fiducial marker positions on the CBCT and subsequent cine portal images. To facilitate marker discrimination, portal images were contrast enhanced using a Laplacian of Gaussian filter with a 3.5-mm kernel (SD, 1.0 mm) (29). Only the first cine image from the first segment of each beam was analyzed because markers tended to be obstructed by MLCs in subsequent segments. To maintain accuracy, EPI images with only 1 identifiable marker were excluded, as were marker localizations with an interobserver error > 1.5 mm. If only 1 marker was obscured by bony anatomy, its portal-imaged location was estimated from its position relative to the other markers on the CBCT scan.

#### Step 2: Autoscan-based prostate localization

The time stamps, prostate motion (translation and rotation) estimates in room coordinates, and *C* values were extracted from Autoscan data log files. To identify couch motion, treatment beam activation, and associated time stamps, linac logs were analyzed. Synchronization between Autoscan and linac was achieved by identifying couch motion timings in both logs. EPI acquisition times were synchronized to the linac by identifying beam-on time stamps. For a given beam, the US-monitored estimate of 3D prostate displacement was identified at the time of the first segment. The initial CBCT marker positions were then shifted by this estimate.

#### Step 3: Comparison between Autoscan and EPI fiducial marker–based prostate motion estimates

The 3D Autoscan estimates of marker position were projected onto the 2D EPI plane for a given beam. Clinically, prostate rotation is incorporated into the Autoscan GPV center-of-mass (CoM) estimates but not displayed to the operator. The GPV CoM could not be accurately identified on CBCT or EPI images, owing to US-CBCT co-registration uncertainties. Instead, the projected markers' CoM was compared with the CoM of the portal-imaged markers, and GPV rotation was disregarded. Monitoring error (*E*) was quantified as the difference between the markers' projected and EPI CoMs in both horizontal and vertical portal image axes (u axis and v axis, respectively).

EPI panel flex < 2 mm with gantry angle may affect the position of features on portal images (30). Flex-induced shifts on each portal image were measured and a rigid 2D correction applied. The MLC geometry for each field was identified from the IMRT plan and used to create a template for each portal image. Flex correction was then applied through a correlation-based template match to the portal image. A gray-level threshold was applied to each portal image to enhance beam edge contrast prior to cross correlation.

### Precision of fiducial marker localization

A phantom study quantified the uncertainty associated with marker localization in the EPI plane. Three fiducial markers were mounted in the treatment field on a radiolucent holder attached to a motion platform. A CBCT scan established initial marker positions. Portal images were acquired for a 5-field IMRT sequence. The platform was moved between fields, approximating the magnitude of intrafraction prostate motion (up to 5 mm in all directions). Resulting portal image marker CoM measurements were compared with the projected CoM derived from shifting the initial CBCT marker positions by the programmed motion. The experiment was repeated 6 times, with repositioning of the gantry and imaging panels to emulate typical interfraction variation. The median 2D error $\left({\tilde{E}}_{phan}\right)$ and 95% limit of agreement (LOA) were calculated for a total of 30 images across all 6 fractions.

### Influence of fiducial markers and image quality on Autoscan motion estimation

To assess the influence of the presence of fiducial markers and image quality (IQ) on Autoscan performance, 3 observers (2 physicists and 1 therapist) reviewed all Guide and CBCT images analyzed for this study using Clarity Automated Fusion and Contouring software.

US IQ within a contour grown 2 mm from the GPV was assessed on a 4-point scale using the following criteria: 1, poor, with no discernible features and no prostate boundary; 2, few features and partial boundary; 3, features and partial boundary; and 4, many features and clear boundary. The number of discernible features (*N*) was categorized as either ≤5, >5 but <10, or ≥10.

**Fig. 2:**
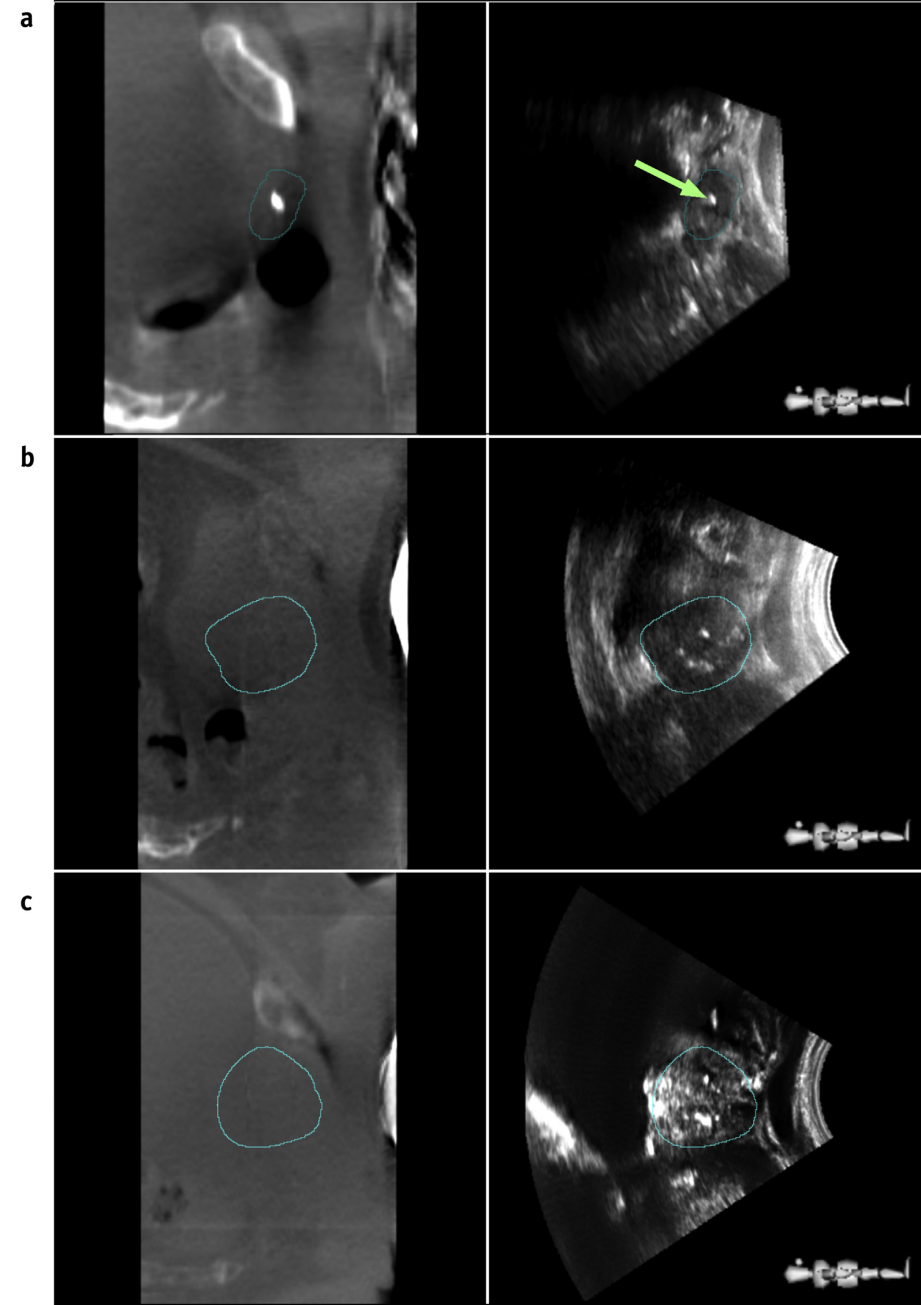
a, Sagittal cone beam computed tomography image with associated transperineal ultrasound image depicting prostate guidance positioning volume contour (blue) and fiducial marker identified visually (green arrow). b, c, Guidance positioning volume features visible on transperineal ultrasound images but not cone beam computed tomography images. High-intensity (white) ultrasound features seen in the central region of the prostate likely are calcifications. Ultrasound features are also observed near the urethra and bladder-prostate interface. (A color version of this figure is available at https://doi.org/10.1016/j.ijrobp.2018.04.008.)

The 3 most prominent US features within each GPV were identified. Fiducial marker positions were also located on CBCT images. Feature and fiducial marker locations were compared. Figure 2 illustrates features visible on both CBCT and TPUS images for 3 typical patients with mean monitoring confidence values between 0.89 and 0.94. A US image feature was deemed to be a fiducial marker if it was identified by all 3 observers to ≤ 3 mm of the mean marker position identified on the registered CBCT image. Observers reviewed Guide and CBCT images in separate sessions to blind them to the feature positions when identifying marker positions.

### Data analysis

Error distributions were tested for normality using a 1-sample, nonparametric Kolmogorov-Smirnov test. Bland-Altman plots with nonparametric 95% LOAs were used to assess the agreement between EPI-based and Autoscan-based prostate motion (31). Autoscan monitoring confidence (*C*) exhibited a range of C ≥ 0 but ≤ 1, warning the user if *C* was <0.5 so that the user could pause treatment and reposition the patient. The relationship between *C* and *E* was investigated using linear regression. Linear regression was also used to investigate whether GPV rotation was associated with *E.*

Guide images were divided into 2 groups, marker positive and marker negative, based on whether markers were identifiable on US images. To ascertain whether markers influenced monitoring performance, mean *C* and *E* per fraction (*C*_*f*_ and *E*_*f*_, respectively) were calculated and their medians (${\tilde{C}}_{f}$ and ${\tilde{E}}_{f}$, respectively) recorded for each group.

Guide images were also classified according to their IQ and categorization of the number of discernible features (*N*). The distributions of *C*_*f*_ and *E*_*f*_ in each category were examined to determine whether monitoring performance changed with either IQ or *N*. Box plots of *C*_*f*_ and *E*_*f*_ were calculated for each category. Kruskal-Wallis 1-way analysis of variance (ANOVA) tests were also used to ascertain whether distributions in each category were statistically different from each other. All image processing and statistical analysis were performed in MATLAB (2016a release; The MathWorks, Natick, MA).

## Results

### Patient data

A total of 352 portal images (the first from each field) and corresponding Autoscan data from 80 fractions were analyzed across 16 patients. Of these portal images, 17 were excluded owing to large interobserver variation of marker localizations. Intrafraction motion estimates throughout 1 fraction were consistently below the Clarity-defined threshold of *C* < 0.5 and also excluded. This left 330 fields with usable data.

### Comparison of Autoscan- versus marker-measured motion

**Fig. 3:**
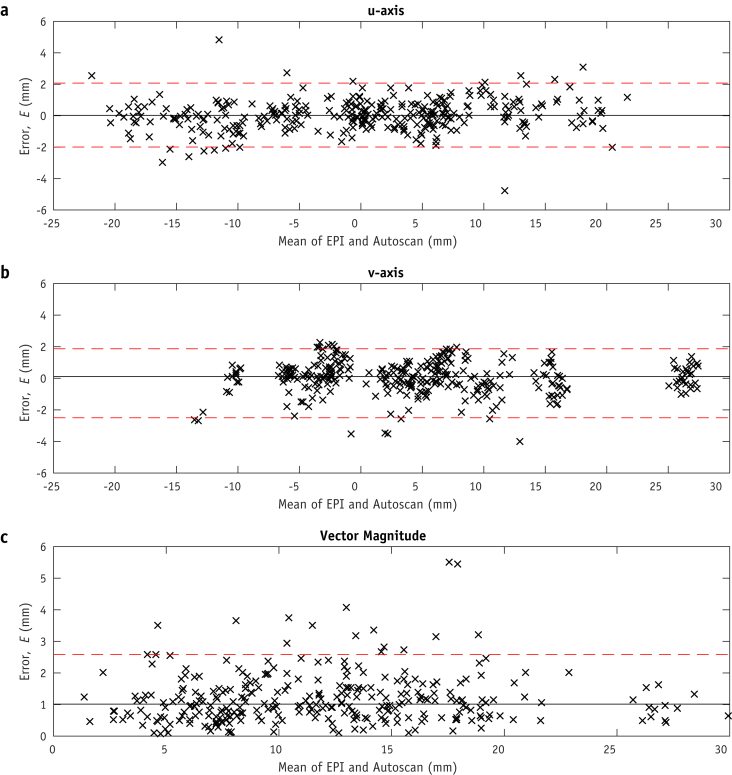
Bland-Altman plots of Autoscan error (*E*), depicted on the plots' y axes, as the difference between monitoring and electronic portal imaging (EPI) estimates of prostate position. Median *E* is shown by the solid line, and 95% limits of agreement (LOAs) are shown by the dashed lines: in the portal image horizontal u axis, with a median of 0 mm and 95% LOAs of –2.0 to 2.1 mm (a); vertical v axis, with a median of 0.1 mm and 95% LOAs of –2.5 to 1.9 mm (b); and Bland-Altman plot of the 2-dimensional error vector magnitude $\left(\sqrt{u^{2}+v^{2}}\right)$, with a median of 1.0 mm and 95% LOA of 2.6 mm (c).

Errors were nonnormally distributed in the vertical v-axis (*P* = .108 for u axis, *P* = .038 for v axis). Table 1 shows 25%, 50%, 75%, and 95% LOAs. The median horizontal u-axis error was ${\tilde{E}}_{u}\;=\;0.0$ mm (95% LOAs, −2.0 to 2.1 mm). The median v-axis error was ${\tilde{E}}_{v}\;=\;0.1$ mm (95% LOAs, –2.5 to 1.9 mm). For both the u and v axes, the median absolute error was 0.6 mm. The median 2D error vector magnitude was ${\tilde{E}}_{\left(u,v\right)}\;=\;1.0$ mm (2.6 mm). Bland-Altman plots in Figure 3 show the distribution of errors (*E*). From the phantom study, the median experimental error measured ${\tilde{E}}_{plan\;}=\;0.8$ mm (95% LOA, 1.1 mm) for 30 measurements over 6 simulated fractions.

**Table 1:** Limits of agreement for monitoring errors (*E*) depicted in Figure 3 (Bland-Altman plot)

Limit of agreement	u-Axis, mm	v-Axis, mm	2D magnitude, mm
25%	−0.2 to 0.3	−0.2 to 0.4	0.6
50%	−0.5 to 0.6	−0.5 to 0.7	1.0
75%	−0.9 to 1.0	−1.1 to 1.1	1.5
95%	−2.0 to 2.1	−2.5 to 1.9	2.6
$\left|\tilde{E}\right|$	0.6	0.6	1.0

*Abbreviation:* 2D = 2-dimensional.

The median absolute error, $\left|\tilde{E}\right|$, is 0.6 mm for both axes and 1.0 mm for the 2D magnitude.

Linear regression results are shown in Figure E1 (available online at https://doi.org/10.1016/j.ijrobp.2018.04.008). Proprietary monitoring confidence (*C*) relative to *E* showed no linear relationship between these quantities (*R*^2^ = 0.03) or between the GPV motion or rotation and *C* (*R*^2^ ≤ 0.11) or *E* (*R*^2^ ≤ 0.06).

### Influence of fiducial markers, features, and IQ on Clarity Autoscan motion estimation

Table 2 lists the number of markers identified by each observer per fraction and per patient. Only 3 of a possible 240 markers were unanimously identified. This consensus was achieved in only 2 fractions from 1 patient. Multiple US image features were consistently identified within the Guide GPV, including the 2 marker-positive fractions with *N* > 5 but < 10.

**Table 2:** Number of markers identified on ultrasound images by observers, with *N* and IQ per fraction and median *C*_*f*_ and *E*_*f*_ for marker–identifiable (positive) and marker–unidentifiable (negative) groups

	Observer 1		Observer 2		Observer 3		All observers	
	Marker positive	Marker negative	Marker positive	Marker negative	Marker positive	Marker negative	Marker positive	Marker negative
Fiducial markers	8	232	3	237	9	231	3	237
Fractions	6	74	2	78	7	73	2	78
Patients	3	14	1	16	4	13	1	16
*N*								
≤5	1	16	0	12	3	45	0	18
>5 but <10	3	24	2	16	4	28	2	32
≥10	2	34	0	40	0	0	0	28
IQ score								
1	0	2	0	0	0	0	0	0
2	1	19	0	23	1	28	0	23
3	5	35	1	26	6	29	2	37
4	0	18	1	29	0	16	0	18
Confidence (median *C*_*f*_)	0.90	0.87	0.84	0.87	0.87	0.87	0.84	0.87
Error (median *E*_*f*_), mm	0.69	1.02	0.69	1.02	0.84	1.07	0.69	1.02

*Abbreviations: C*_*f*_ = confidence factor per fraction; *E*_*f*_ = monitoring error per fraction; IQ = image quality; *N* = number of discernible features.

Kruskal-Wallis ANOVA tests indicated no statistically significant difference between the *C*_*f*_ distributions across IQ scores. Likewise, *C*_*f*_ distributions were statistically indistinguishable across the different *N* categories. Even so, median *C*_*f*_ consistently improved with increasing IQ score and *N*. No statistically significant difference was found between the *E*_*f*_ distributions across IQ scores. Median *E*_*f*_ also consistently improved with increasing IQ score and *N*. Box plots and full ANOVA results are given in Figure E2 (available online at https://doi.org/10.1016/j.ijrobp.2018.04.008). Additional analysis of error distributions relative to the left-right, anterior-posterior and superior-inferior patient axes are provided in Appendix E1 (available online at https://doi.org/10.1016/j.ijrobp.2018.04.008).

## Discussion

The median absolute difference between Autoscan-predicted and portal-imaged prostate marker positions was 0.6 mm, with 95% of differences ≤ 2.5 mm in the EPI u and v axes scaled to the isocenter. This finding is in agreement with a previous conference contribution that reported a mean difference between EPI and Clarity of 1.2 mm (SD, 1.0 mm) across 4 patients (32). Our study found similar agreement to that demonstrated for other motion-monitoring systems validated in vivo using radiographic imaging. RayPilot intrafraction prostate motion was approximated by moving the transponder inside the prostatic urethra. The mean and maximum 3D errors were 1.7 mm (SD, 1.0 mm) and 4.6 mm, respectively (18). Only Calypso interfraction motion estimation validations have been reported 16, 33. Willoughby et al (16) reported mean and maximum 3D errors of 1.5 mm (SD, 1.1 mm) and 3.8 mm, respectively, over 44 fractions in 11 patients. Ogunleye et al (33) found mean differences of 1.2 mm (SD, 0.9 mm), 1.1 mm (SD, 0.9 mm), and 0.7 mm (SD, 0.5 mm) in the anterior-posterior, superior-inferior, and left-right directions, respectively. Foster et al (34) found mean differences of –0.81 mm (95% LOAs, −2.83 to 2.67 mm), –0.41 mm (95% LOAs, −3.41 to 2.59 mm), and 0.06 mm (95% LOAs, −1.97 to 2.09 mm) for the anterior-posterior, superior-inferior, and left-right directions, respectively, over 250 fractions.

The KIM system was evaluated in vitro, finding a mean positional error over 6 prostate-derived motion traces of 0.6 mm (SD, 0.4 mm) for left-right, 0.2 mm (SD, 0.1 mm) for superior-inferior, and 0.4 mm (SD, 0.4 mm) for anterior-posterior (35). KIM has not been compared with any other 4-dimensional localization technique in vivo.

We propose that in a prostate SBRT setting, both Clarity and CBCT will be used. CBCT will be used for daily interfraction adaptation. Clarity Autoscan will measure intrafraction motion for use with dMLC tracking or gating, which has been shown to improve target dose coverage 24, 36. Several comparable motion compensation methods have been shown to reduce planned therapy γ-failure rates for a sample of representative prostate motions from 17.3% to 1.4% 37, 38. The feasibility of using Clarity with dMLC tracking has been previously described by Fast et al (24) with a similar dosimetric advantage to other methods. Owing to their similar accuracy, recommendations for margin reduction using Calypso may be applied to Clarity. Tanyi et al (39) used Calypso with a gating threshold of 4 mm for shifts > 1 second, calculating a reduced PTV margin of 1.4 mm, 2.3 mm, and 2.6 mm (left-right, anterior-posterior, and superior-inferior, respectively) using the methodology of van Herk et al (40). Keall et al (15) reported dMLC tracking using Calypso in vivo for prostate SBRT. PTV margins were not changed, however, and the authors advised caution when considering margin reduction until further clinical data are collected.

Clinically, poor IQ can arise from inadequate probe placement; shadowing of features obstructed by bony anatomy, such as the pubic symphysis; or attenuation from excess subcutaneous perineal tissue. This may limit Autoscan confidence and degrade monitoring accuracy. In this study, only a single fraction exhibited confidence factors consistently below the Clarity alert threshold, *C* < 0.5, owing to a poorly positioned probe. Poor confidence scores were avoided by following an imaging protocol to ensure consistent patient setup relative to SIM, checking that the field of view fully encompassed the GPV with the prostate centered. RT radiographers were trained to optimize these setup parameters in addition to their routine clinical duties. Only 1 fraction out of 80 exhibited a low monitoring confidence, where it was deemed the protocol was not adequately followed.

This study found no relationship between monitoring performance (*C*_*f*_, *E*_*f*_) and IQ (IQ score, *N*). However, a pre-fraction monitoring session to manually inspect the Autoscan confidence factor may be used to ensure that *C* is >0.5 to avoid suspending treatment. No patients in this study exhibited consistently poor motion estimates across all fractions; however, the aforementioned preselection step could also be implemented at SIM to ensure the patient is eligible for Autoscan use.

Some experimental limitations of the study were identified. The 3 largest monitoring errors in figure 3c [*E* ≥ 4.1 mm] were attributable to the position uncertainty arising from different Guide and CBCT acquisition times and inaccurate synchronization between monitoring and portal EPI acquisition. The Guide scan typically lasted < 3 seconds, recorded at the start of the 60-second CBCT acquisition. Once completed, the RPV was manually positioned in Guide as a reference for the subsequent monitoring session (GPV). This did not account for prostate motion during most of the CBCT scan, which has been identified as a source of error in previous studies comparing Calypso with CBCT (34). The 2 largest monitoring errors where *E*_*(u, v)*_ was >5 mm were attributable to motion during CBCT. This was verified by manually registering CBCT and Guide images, indicating a prostate shift of 6 mm. As this magnitude of motion was rarely observed and it risked introducing greater errors, manual registration was not conducted.

Monitoring traces were synchronized to beam event messages in the linac log in lieu of an EPI acquisition time stamp. This indirect synchronization between monitoring and portal imaging could have produced further uncertainty, which could not be quantified; however, an 8-mm anterior prostate displacement was observed immediately after a segment with an error *E*_*(u, v)*_ = 4.1 mm.

Interobserver variation of marker localizations was identified as a potential source of error, particularly on CBCT, where spatial resolution is poor (34). This was mitigated by excluding localizations with variation > 1.5 mm. Disregarding prostate rotation was another potential source of error; however, rotations are not provided to the operator during clinical use, and no relationship between rotation and *E* was identified.

Monitoring confidence improves with US image feature density (28). Intrinsic anatomic features were more frequently observed than implanted fiducial markers on TPUS images. Markers were discernible for only 2 fractions, in which the average number of features was high: *N* > 5 but < 10 (Fig. 2). As a result, fiducial markers were not considered to significantly influence Autoscan confidence (*C*).

## Conclusions

The Clarity system can monitor intrafraction prostate motion with an accuracy in the 2D EPI frame of reference of 0.6 mm (median absolute monitoring error), with 95% LOAs between −2.5 and 2.1 mm, which is comparable with other motion-monitoring systems used in RT. Autoscan motion estimation confidence was not reliant on the presence of intraprostatic markers, and no significant relationship between estimation confidence and IQ was observed.
