# Supplementary material for: In Vivo Validation of Elekta's Clarity Autoscan for Ultrasound-based Intrafraction Motion Estimation of the Prostate During Radiation Therapy
Source: Int J Radiat Oncol Biol Phys. 2018 Nov 15;102(4):912–21. doi: 10.1016/j.ijrobp.2018.04.008 (PMC6202949; doi:10.1016/j.ijrobp.2018.04.008)
Supplement: Supplementary Material 1 [file mmc1.docx]

SUPPLEMENTARY MATERIALS 1


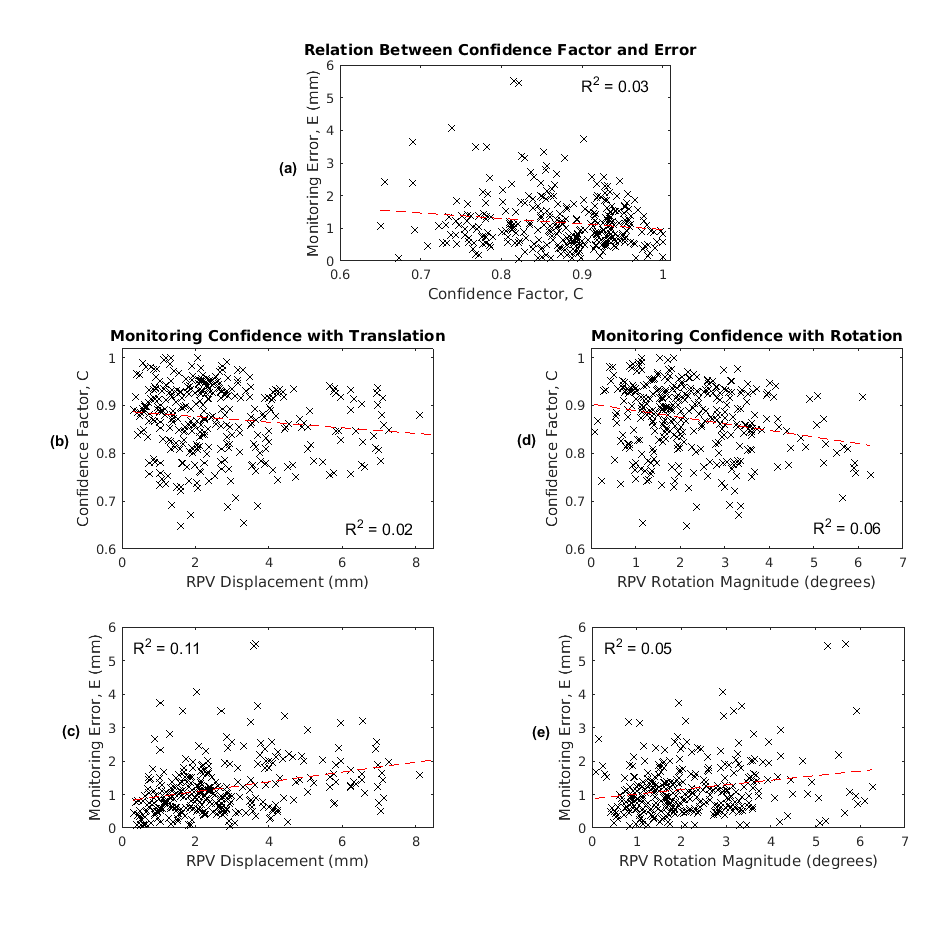


Figure SM1. Linear regressions of: (a) Confidence factor, *C*, with Monitoring error, *E*; (b-c) *C* and *E* relative to prostate translation; (d-e) *C* and *E* relative to prostate rotation.
