# Supplementary material for: In Vivo Validation of Elekta's Clarity Autoscan for Ultrasound-based Intrafraction Motion Estimation of the Prostate During Radiation Therapy
Source: Int J Radiat Oncol Biol Phys. 2018 Nov 15;102(4):912–21. doi: 10.1016/j.ijrobp.2018.04.008 (PMC6202949; doi:10.1016/j.ijrobp.2018.04.008)
Supplement: Supplementary Material 2 [file mmc2.docx]

SUPPLEMENTARY MATERIALS 2.


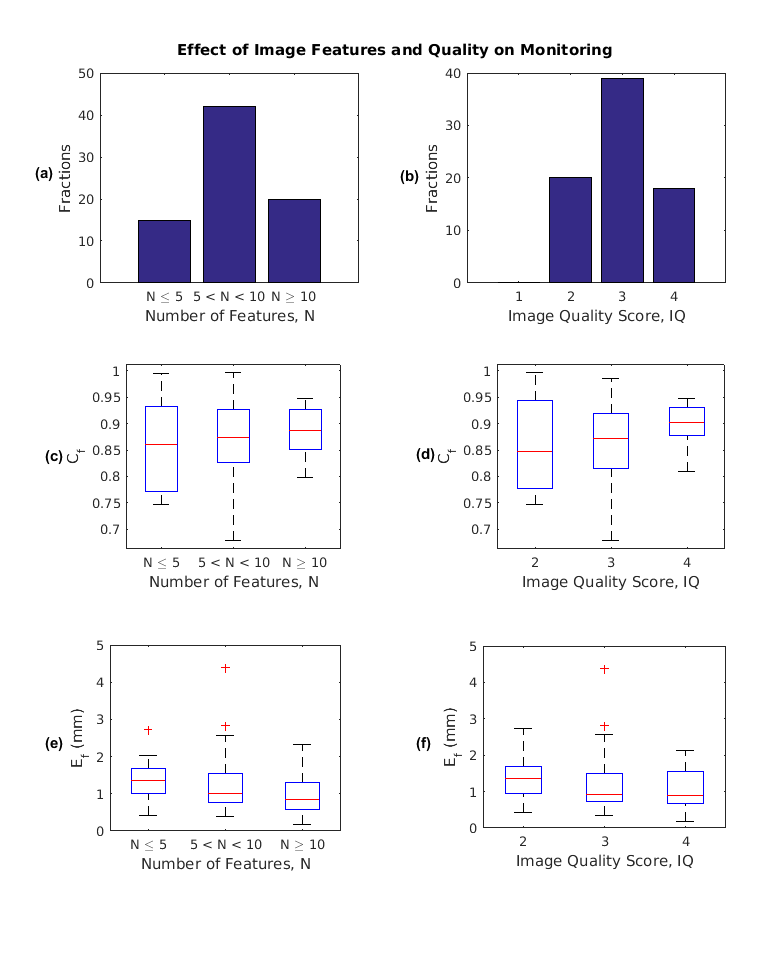


Figure SM2. Histograms of number of fractions categorised by number of features, *N* (a) and image quality score, *IQ* (b). Boxplots of the fractions’ mean Confidence factor, *C_f_*, distributions by *N* (c) and *IQ* (d), with ANOVA results *p* = 0.547 and *p* = 0.222 respectively. Boxplots of the fractions’ mean difference between Clarity and EPI derived estimates, *Ef*, by *N* (e) and *IQ* (f), with ANOVA results *p* = 0.0673 and *p* = 0.324.
