# Supplementary material for: In Vivo Validation of Elekta's Clarity Autoscan for Ultrasound-based Intrafraction Motion Estimation of the Prostate During Radiation Therapy
Source: Int J Radiat Oncol Biol Phys. 2018 Nov 15;102(4):912–21. doi: 10.1016/j.ijrobp.2018.04.008 (PMC6202949; doi:10.1016/j.ijrobp.2018.04.008)
Supplement: Supplementary Material 3 [file mmc3.docx]

SUPPLEMENTARY MATERIALS 3

The Autoscan system reconstructs a 3D volumetric image from a mechanically swept transducer. When using the transperineal probe, 2D B-mode images are acquired in the sagittal plane and the transducer is swept along the transaxial plane to create a 3D image. Monitoring accuracy is lower in the left-right axis owing to sparser spatial sampling. To ascertain whether prostate motion estimation accuracy was affected, portal images acquired within +/-5° of the major room axes were collected from the original 352. For gantry angles close to 0° or 180°, it was assumed the portal image axes align to the Autoscan axes along the left-right (LR) and superior-inferior (SI) directions. For gantry angles close to 90° and 270°, it is assumed the portal image axes align to the anterior-posterior (AP) and SI Autoscan axes. The errors, *E*, defined as the difference between CBCT and Autoscan prostate motion estimates were grouped by axis.

One sample Kolmogorov-Smirnov tests showed error distributions were non-normal for the AP and SI axes (*p* = 0.52 and 0.45 respectively). The LR error distribution was normal (*p* = 0.04).

Table SM3 lists the percentile ranges and 95% limits of agreement (LOA) of the absolute errors, *|E|*, for each axis. Histograms of the absolute errors, *|E|*, are plotted in figure SM3. The total number of segments were 76, 144 and 220 in the LR, AP and SI datasets respectively. Median *|E|* was 0.6 mm in all three axes.

The LR 95% LOA is small compared to other axes, suggesting Autoscan performance was not overtly affected by the system’s inherently lower transaxial accuracy. The AP 95% LOA is largest. This axis is associated with the greatest magnitude of prostate motion [1]. It is speculated that larger *|E|* in both the AP and SI directions are due to increased registration uncertainty between CBCT, Autoscan and MV images owing to unaccounted patient motion during CBCT acquisition.

Table SM3. Percentile range of absolute errors, |E|, in each axis.

| **Percentile** | **LR (mm)** | **AP (mm)** | **SI (mm)** |
| --- | --- | --- | --- |
| **25%** | 0.2 | 0.3 | 0.2 |
| **50%** | 0.6 | 0.6 | 0.6 |
| **75%** | 0.8 | 1.2 | 1.1 |
| **95%** | 1.6 | 2.4 | 2.1 |

Figure SM3. Histograms of absolute error, |E|, at gantry angles aligned to the Autoscan axes: (a) Left-right, (b) Anterior-posterior and (c) Superior-inferior.

References

1. Western, C., D. Hristov, and J. Schlosser, *Ultrasound Imaging in Radiation Therapy: From Interfractional to Intrafractional Guidance.* Cureus, 2015. **7**(6): p. 1-19.
